# VDD Pacemaker Implantation Without Fluoroscopy for Complete Heart Block During Pregnancy: A Case Report

**DOI:** 10.21470/1678-9741-2025-0290

**Published:** 2026-09-08

**Authors:** Roberto Tofani Sant’Anna, Tiago Luiz Luz Leiria, Marco Aurélio Lumertz Saffi, João Ricardo Michielin Sant’Anna, Gustavo Glotz De Lima

**Affiliations:** 1 Instituto de Cardiologia do Rio Grande do Sul, Fundação Universitária de Cardiologia, Porto Alegre, Rio Grande do Sul, Brazil; 2 Hospital de Clínicas de Porto Alegre, Porto Alegre, Rio Grande do Sul, Brazil

**Keywords:** Ionizing Radiation, Pregnancy, Atrioventricular Block.

## Abstract

Ionizing radiation is associated with an increased risk of malignancy, especially in fetuses. Therefore, in the rare situation of a pregnant patient in need of a pacemaker, alternatives to conventional fluoroscopy are required. We describe a case of successful implantation of a Ventricle-paced, Dual-sensing, Dual-response pacemaker without fluoroscopy in a 32-year-old female patient using electroanatomic 3D mapping. She had severe bradycardia due to complete atrioventricular block, which impeded intrauterine growth restriction in the fetus.

## INTRODUCTION

**Table:** 

Abbreviations, Acronyms & Symbols	
AV	= Atrioventricular
AVB	= Atrioventricular block
RV	= Right ventricle
SVT	= Supraventricular tachycardia
VDD	= Ventricle-paced, Dual-sensing, Dual-response

Management of bradycardia during pregnancy always poses a dilemma. Pacemaker implantation exposes the fetus to the risks of radiation and anesthetic procedures^[1]^. Conversely, if left untreated, severe bradycardia can lead to hemodynamic instability and can disrupt the normal fetal growth^[2]^. There are some reports on pacemaker implants without fluoroscopy use, some with echocardiography only, others using low-dose X-ray exposure associated with electroanatomic 3D mapping, and a combination of echo-guided with 3D mapping^[3,4]^.

Here, we describe the case of a pregnant patient with complete atrioventricular block (AVB) and intrauterine growth restriction who underwent pacemaker implantation solely based on electroanatomic 3D mapping without any additional imaging modality. In contrast to other reports and case series, by using a Ventricle-paced, Dual-sensing, Dual-response (VDD) lead, we were able to virtually see the pacing lead in almost its totality for better lead deployment.

## CASE PRESENTATION

A 32-year-old female patient with a gestational age of 32 weeks presented with restrictive intrauterine fetal growth due to a complete maternal AVB. On physical examination, there were no remarkable signs other than a cannon A wave on her jugular pulse. She also had no signs or complaints of heart failure. Laboratory test results were negative for lupus, other vasculitis, and infectious diseases that course with AVB. Echocardiogram showed normal left ventricular function and volume. Electrocardiography revealed a complete AVB with an escape rhythm, narrow QRS (41 bpm), and atrial rate of 68 bpm (Figure 1). Fetal echography revealed a single gestational fetus in the 12^th^ percentile for growth and weight for gestational age. These findings led to a decision to implant a permanent pacemaker. We opted to proceed with the operation using a zero-fluoroscopy approach to minimize any potential adverse effects of ionizing radiation on the fetus, even at this gestational age.

**Fig. 1:**
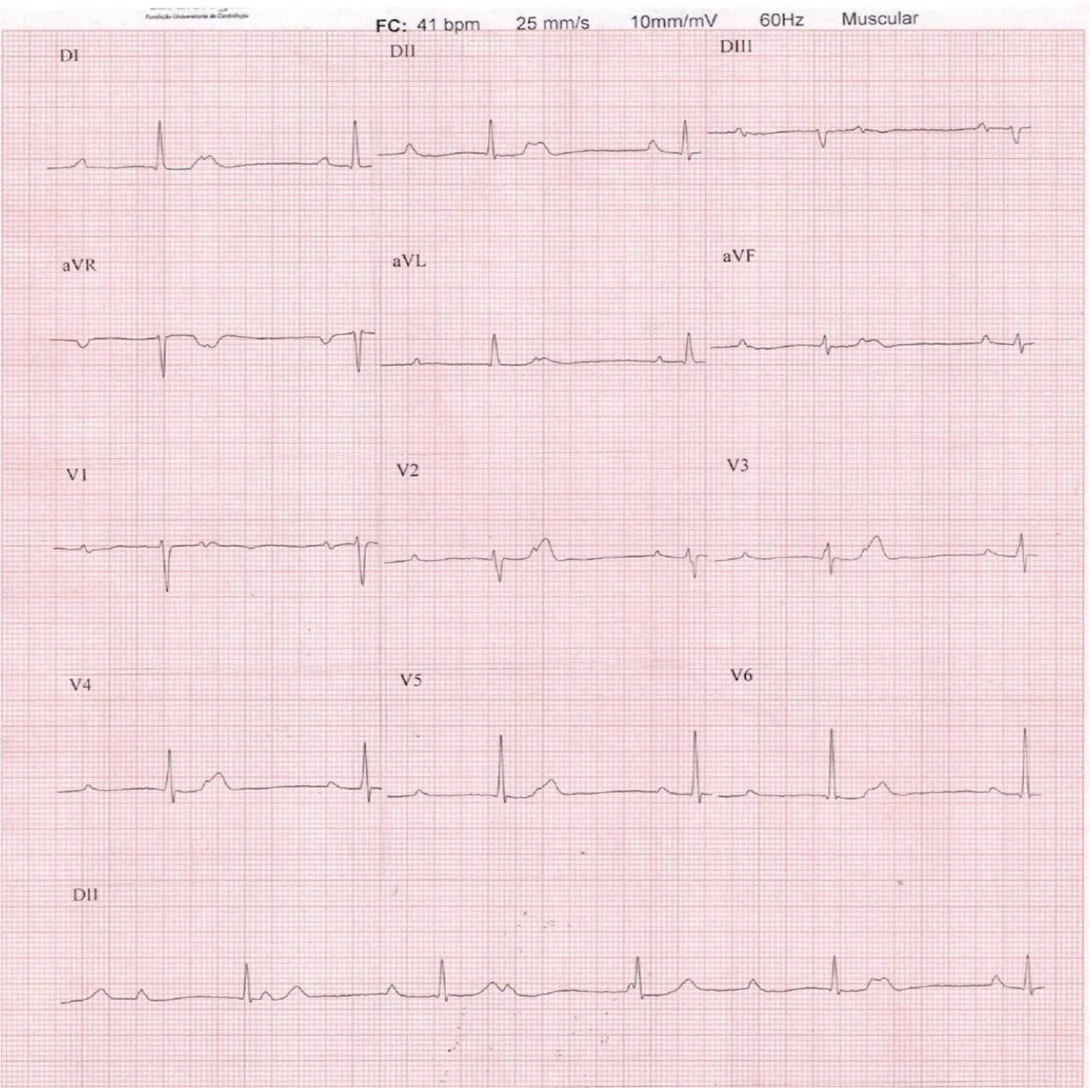
Electrocardiogram exhibiting a complete atrioventricular block with a scape rhythm with a narrow QRS (41 bpm) and an atrial rate of 68 bpm.

### Surgical Technique

The procedure was performed under light sedation and local anesthesia. Briefly, a puncture of left subclavian was performed, and a 7-French sheath was introduced using Seldinger technique. Decapolar catheter (Abbott IBI Inquiry™, 6 Fr, large curve, St. Paul, Minnesota, United States of America) was connected to an EnSite NavX™ system (Abbott/St. Jude Medical, St. Paul, Minnesota, United States of America) to construct the anatomy of the subclavian vein and superior vena cava. We monitored the electrograms obtained using the decapolar catheter to ensure the exact moment that we entered the right atrium. The right atrial geometry was collected, and the His region was mapped leaving a shadow of the catheter to mark the place of the septal atrioventricular (AV) junction to access the right ventricle (RV). Once in the atrium, the sinus node recovery time was measured, and sinus function was normal, which was crucial for selection of the stimulation mode and pacing lead. After completing the veins, right atrial, and right ventricular geometries, we programmed the 3D system to recognize the VDD pacing lead that we selected (AV Plus™ 1368 lead, Abbott/St. Jude Medical, St. Paul, Minnesota, United States of America) (Figure 2), because no atrial pacing would be required in our case, only sensing to ensure AV synchrony. The sheath was then replaced over the wire using a 10-French tear-away sheath for lead implantation. The permanent pacing lead was attached to the 3D system using standard lead testing cables, and a virtual catheter was created using the default specifications of the lead to represent it almost in its entire length on the tridimensional model (15 mm from the tip to the ventricular ring, 110 mm from the tip to the 1^st^ atrial ring, and 130 mm from the tip to the 2^nd^ atrial ring). After introducing the lead on the sheath, we navigated it to the created shadows of the vascular structures until we arrived at the RV. By having four individual rings on it, the VDD lead could be fully visualized on the map, making the navigation more precise and avoiding unnecessary loops. When arriving at the RV, we advanced the lead until we reached the apical position on the geometry, and this position was tagged to compare the initial and final lead positions after peeling the venous sheath. Pacing at that site confirmed the apical placement of the lead (12-lead electrocardiogram with a QRS morphology showing a left bundle branch pattern with a negative QRS on leads III, V1, and V6) (Figure 3). The lead was sutured to the muscular plane, a subcutaneous pocket was created, and the pacing generator was connected to the VDD pacing lead. The total procedure time was 65 minutes with zero X-ray exposure.

**Fig. 2:**
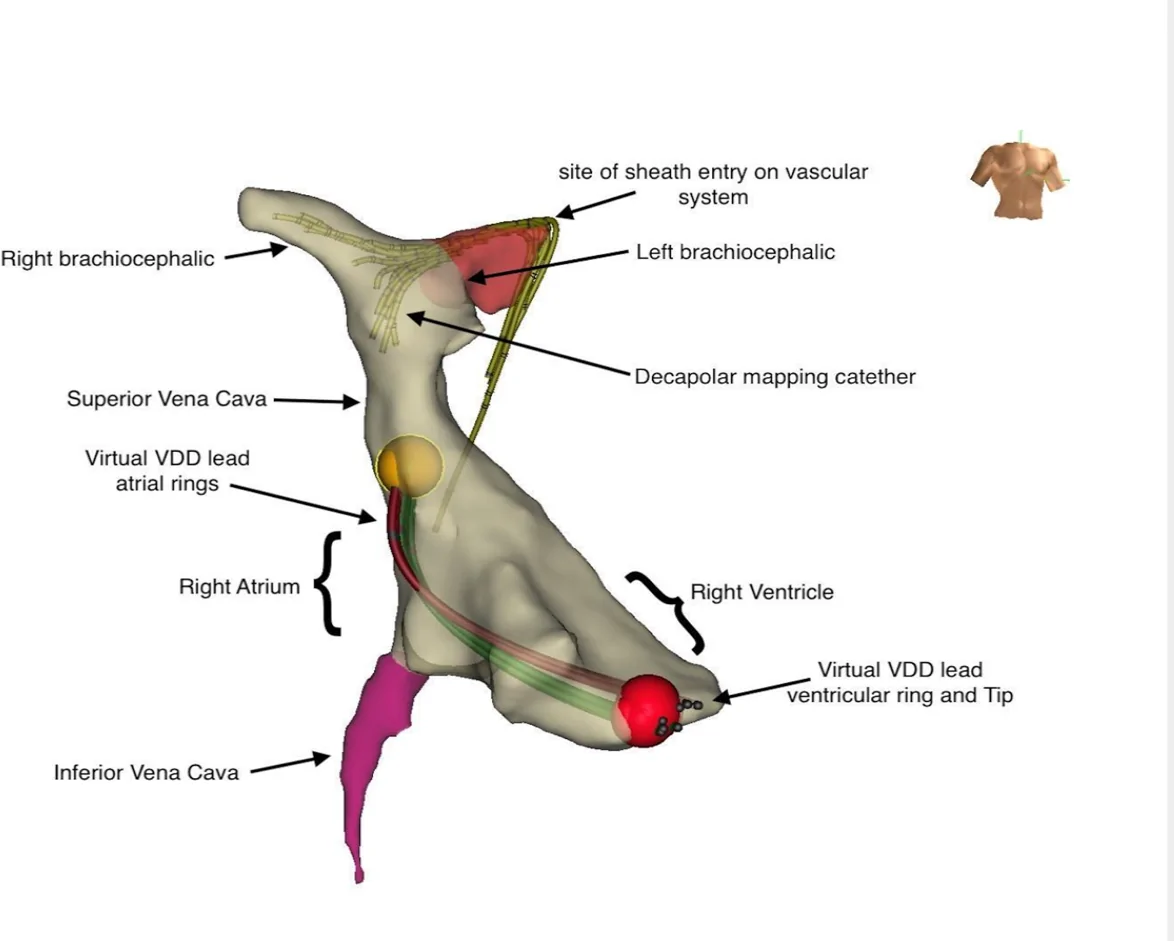
High-resolution electroanatomic 3D map using the EnSite NavX™ system (Abbott/St. Jude Medical, St. Paul, Minnesota, United States of America), demonstrating detailed reconstruction of the superior vena cava, right atrium, and right ventricle. The Ventricle paced Dual sensing Dual response (VDD) lead is fully visualized from its ventricular to atrial rings, with the final apical right ventricular positioning marked and verified.

**Fig. 3:**
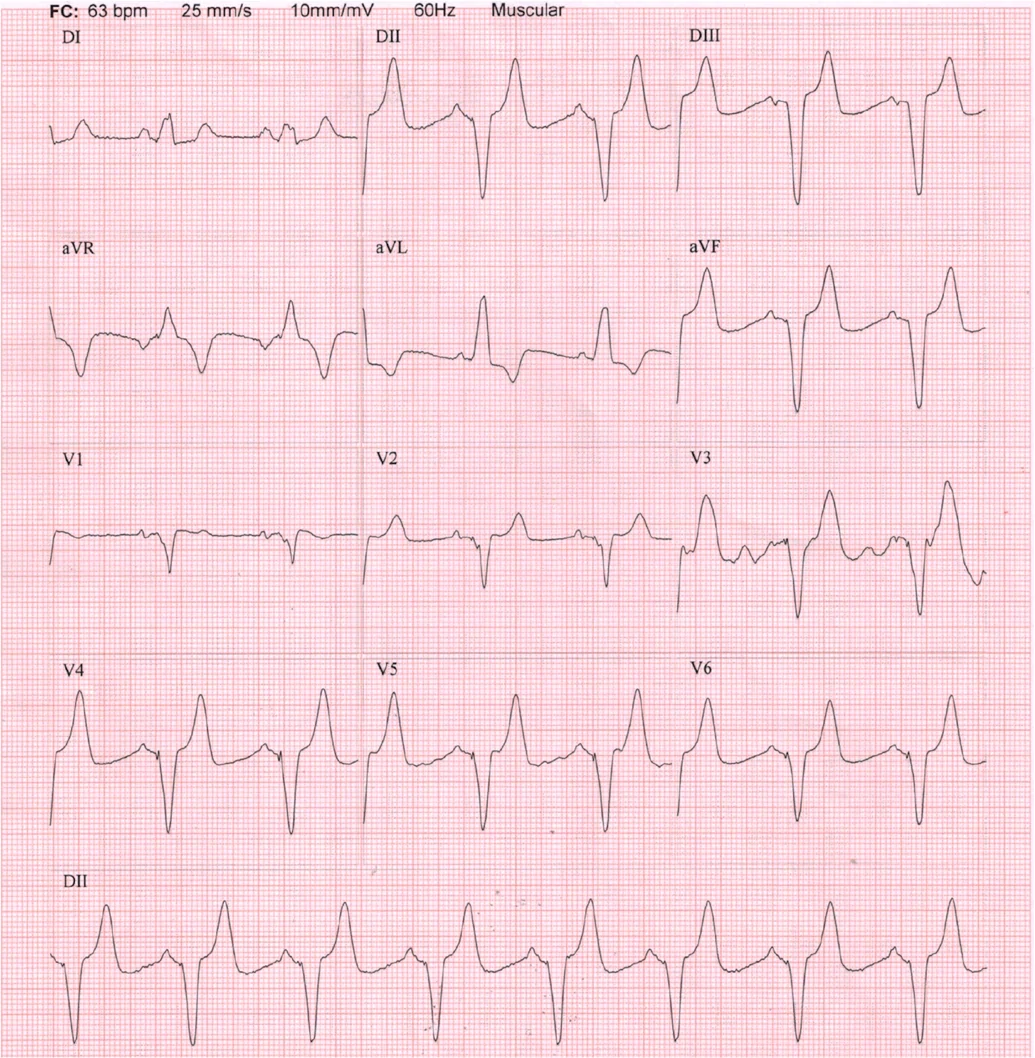
Electrocardiogram with a QRS morphology showing a left bundle branch pattern with a negative QRS on leads III, V1, and V6.

During the procedure, special care was required to navigate the pacing lead solely using the 3D map visualization. Manipulation of the tear-away sheath without fluoroscopic guidance demanded increased reliance on accurate anatomical reconstruction and electrogram feedback. To avoid inadvertent loops or excessive tension, we used shadow-marked geometry and ensured visualization of all electrode rings to confirm linear progression of the lead. These strategies minimized the risk of postoperative lead dislodgement.

The patient tolerated the procedure well and remained clinically stable throughout the remainder of her pregnancy. Delivery occurred via cesarean section at 37 weeks of gestation due to obstetric indications, and the newborn had appropriate weight and Apgar scores. Follow-up at three months postpartum confirmed appropriate device function and no signs of lead dislodgement or complications.

## DISCUSSION

Radiation can generate specific adverse effects on the fetus during pregnancy, including growth restriction, prenatal death, organ malformation, and intelligence impairment^[1,5]^. Adverse events of this extent usually occur with higher radiation doses than those delivered by fluoroscopy alone. Observational data show that the mean radiation dose for a simple pacemaker insertion is approximately 1 mSv^[6]^. At the described gestational age, this amount of radiation, with the use of appropriate shielding of the fetus, is probably safe, but the risk of long-term adverse events in the offspring cannot be ruled out. Therefore, in our opinion, during the gestational period, no radiation was better than any radiation. We have also published our experience with zero X-ray exposure during the gestational period for syncopal supraventricular tachycardia (SVT) and our randomized data on SVT ablation^[7,8]^.

While fetal exposure below 50 mGy is generally considered safe, and typical pacemaker implantation results in radiation doses around 1 mGy, the cumulative and long-term risks to the fetus remain incompletely understood. Therefore, even minimal radiation exposure is best avoided when feasible^[9]^. Nonetheless, we acknowledge that in centers without access to electroanatomic mapping systems, a conventional approach using fluoroscopy with abdominal lead shielding could be considered a safe and reasonable alternative. Including this trade-off enhances procedural decision-making in resource-variable settings.

The electroanatomical systems used for cardiac electrophysiology procedures have the advantage of creating the anatomy of the vessel and heart structures by careful manipulation of the catheter inside the circulatory system. EnSite NavX™ system (Abbott/St. Jude Medical, St. Paul, Minnesota, United States of America) uses different sensed impedances and voltage gradients with real-time computing for the creation of anatomical, activation, and voltage maps. The system can calculate the 3D position of each electrode present in any multipolar catheter and represent them in a virtual 3D model. This system feature made it possible for us to recreate the AV Plus™ 1368 VDD pacing lead on almost its entire length. In our opinion, this is crucial for lead placement without any other imaging modality. By visualizing the proximal atrial rings and distal ventricular pacing poles, we were able to navigate the lead to avoid any unintentional loops and curves that could potentially increase the risk of lead dislodgment in the postoperative period. The use of bipolar conventional pacing electrodes, described in most reports^[3,4]^, does not allow the implanter to visualize the lead itself because only the bipolar tip of the electrode pairs can be displayed on the 3D system.

While cost was not the primary focus of this case report, it is important to acknowledge that electroanatomic mapping systems entail a considerable upfront investment compared with conventional fluoroscopy-guided implantation. Nevertheless, their use may be justified by significant clinical advantages, including the elimination of radiation exposure, enhanced accuracy in lead positioning, and a potential reduction in procedure-related complications. These benefits are particularly relevant in complex scenarios such as pregnancy, where the avoidance of fetal radiation exposure provides a compelling rationale that outweighs the additional costs. However, the cost-benefit ratio should always be considered in the broader context of patient safety and procedural precision. In resource-limited settings, conventional fluoroscopy with appropriate abdominal shielding remains a safe and reasonable alternative^[10]^.

## CONCLUSION

Pacemaker implantation during pregnancy with zero radiation has already proven to be a feasible procedure. In our report, the presence of normal sinus node function and the use of VDD pacing lead to ensure AV sequential pacing made it possible to fully visualize the lead for better positioning, ensuring safer and more physiological stimulation.

## Data Availability

The authors declare that the data will be available upon request to the authors.

## Artificial Intelligence Usage

The authors declare use of ChatGPT (OpenAI) for grammar review and language editing. The content produced by the artificial intelligence tool was revised and edited by the authors as necessary, and they take full responsibility for the content to be published.
